# Spatiotemporal Co-existence of Female Thyroid and Breast Cancers in Hangzhou, China

**DOI:** 10.1038/srep28524

**Published:** 2016-06-24

**Authors:** Xufeng Fei, George Christakos, Zhaohan Lou, Yanjun Ren, Qingmin Liu, Jiaping Wu

**Affiliations:** 1 College of Environmental and Resource Sciences, Zhejiang University, Hangzhou, China; 2 Institute of Islands and Coastal Ecosystems, Zhejiang University, Zhoushan, China; 3 Department of Geography, San Diego State University, San Diego, CA, USA; 4 Hangzhou Center for Disease Control and Prevention, Hangzhou, China

## Abstract

Thyroid and breast cancers (TC, BC) are common female malignant tumors worldwide. Studies suggest that TC patients have a higher BC risk, and vice versa. However, it has not been investigated quantitatively if there is an association between the space-time TC and BC incidence distributions at the population level. This work aims to answer this question. 5358 TC and 8784 BC (female) cases were diagnosed in Hangzhou (China, 2008–2012). Pearson and Spearman rank correlation coefficients of the TC and BC incidences were high, and their patterns were geographically similar. The spatiotemporal co-existence of TC and BC distributions was investigated using the integrative disease predictability (IDP) criterion: if TC-BC association is part of the disease mapping knowledge bases, it should yield improved space-time incidence predictions. Improved TC (BC) incidence predictions were generated when integrating both TC and BC data than when using only TC (BC) data. IDP consistently demonstrated the spatiotemporal co-existence of TC and BC distributions throughout Hangzhou (2008–2012), which means that when the population experiences high incidences of one kind of cancer attention should be paid to the other kind of cancer too. The strength of TC-BC association was measured by the IDP coefficients and incidence prediction accuracy.

*Breast cancer* (BC) is the most common malignant tumor among women, especially for women aged between 40 and 60 years old^1^,^2^,^3^,^4^. The distribution of BC shows different degrees of variability, at the local or the global scale^4^,^5^. The BC incidence in developed countries is about 3 times higher than in developing countries, like China^6^. It has been estimated that more than 1.6 million of new BC cases were diagnosed in 2012 worldwide causing over a half million deaths^7^. Many previous studies have discussed the BC risk factors, such as body mass index (BMI), family history of BC, dietary patterns, socioeconomic status, smoking and alcohol consumption^8^,^9^,^10^,^11^,^12^,^13^. Among them, the reproductive, menstrual and hormonal factors (such as parity rate, pregnancy, age at menarche, menstrual cycle regularity, menopausal status, exogenous hormone use and hormone replacement therapy) have been found to have significant impacts on BC risk^14^,^15^,^16^,^17^.

*Thyroid cancer* (TC), on the other hand, is the most common endocrine malignant tumor, and its incidence has displayed a sharp increase during the last few decades^18^. Established TC pathogenic factors are ionizing radiation^19^ and benign proliferative thyroid disease^20^. Other potential factors include vitamins and minerals, diet, fish and shellfish consumption, vegetable consumption, smoking, alcohol intake, ethnic background, genetic factors, higher BMI, and diabetes^21^,^22^,^23^,^24^,^25^,^26^,^27^,^28^,^29^. Some studies have considered the relationship between iodine intake and TC risk, but their results are rather inconclusive^20^,^30^,^31^. Endocrine disrupting compounds such as polychlorinated biphenyls (PCBs), polybrominated diphenyl ethers (PBDEs) and pesticides, which can disturb thyroid function, have been linked to carcinogenesis through a variety of mechanisms^32^,^33^,^34^. TC is gender-biased, i.e., it is more common among females than among males with a female to male ratio of about 3 to 1 in the general population^35^. TC and BC have similar age-specific incidence rates: women between 40–60 years old have the highest TC incidence rate, which is also the case of BC incidence. None of the risk factors mentioned above can account for the TC incidence disparity among different genders. Furthermore, it has been suggested that improved detection and the use of thyroid imaging (leading to an increasing number of detected small papillary cancer cases) may be responsible for the fast increase of reported TC incidences^36^,^37^,^38^. However, not all studies agree with this explanation, arguing, instead, that the increasing number of all-sizes TC observed may be due to two coexisting reasons: increased detection, and environmental risk factors^38^,^39^.

In light of the above studies, it is obvious that BC and TC have certain risk factors in common (genes, lifestyle, diet habits, over-diagnosed and hormonal, menstrual and reproductive factors), in which case one may justifiably hypothesize that the TC and BC distributions are co-existent. Indeed, several researchers have suggested that due to common risk factors there may exist a strong association between TC and BC incidence^40^,^41^.

Generally, the rigorous investigation of potential associations between diseases is a very complicated issue, leading to several criteria for testing possible associations, including deterministic criteria (i.e., necessary and sufficient associations) and stochastic criteria (concerned with factors that raise the chances of certain associations)^3^,^32^,^42^,^43^. The deterministic viewpoint of disease association may be valid only if all the relevant physical and biological laws linking the diseases are known in detail (then, in principle, causality could be established by analyzing the ways in which these laws are formulated and the ways they are verified or rejected by experimentation). Although very desirable, such an approach is rarely materialized in health practice.

Instead, most studies are actually concerned with stochastic disease associations at the population level. While stochastic associations do not usually imply necessary and sufficient causation criteria, they offer useful insight into such a complicated situation of considerable public health significance^32^,^44^. Studies of disease associations in human populations involve a variety of techniques, including visual comparisons of the patterns exhibited by the disease factors, statistical analysis of population disease occurrences across a geographic area, multi-perspective analysis, and geographical information systems techniques^30^,^45^,^46^,^47^,^48^,^49^. Yet, these techniques may suffer certain drawbacks when seeking to identify disease interactions and causation^41^,^43^,^50^.

In this work, by taking into consideration that the assessment of the spatiotemporal variation of disease incidence could improve our understanding of TC-BC associations at the population level, the combination of the *Bayesian maximum entropy* (BME) theory^51^ and the *integrative disease predictability* (IDP) criterion^52^,^53^ provides a powerful quantitative approach to study the spatiotemporal co-existence of TC and BC in Hangzhou city. Specifically, the IDP criterion was used to investigate the spatiotemporal co-existence of TC and BC incidence distributions and assess quantitatively the strength of the TC-BC association in terms of the predictability of the corresponding BME-generated cancer maps.

## Materials and Methods

### TC and BC Incidence Data

Newly diagnosed female TC cases (International Classification of Disease: ICD-10 code C73) and female BC cases (International Classification of Disease: ICD-10 code C50) in the city of Hangzhou (HZ) from Jan 1, 2008 to Dec 31, 2012 were obtained from the Hangzhou center for Disease Control and Prevention (CDC). Detailed information about TC and BC in the study area can be found in the relevant literature^54^,^55^. Briefly, the CDC confirmed 5358 female TC patients and 8784 female BC patients in 8.70 million person-years. According to the detailed residential information available, all cases were allocated to 200 Hangzhou townships. The indirectly standardized ratio of female TC over female BC incidences^56^ was calculated based on female township-level age-specific population data and female TC and BC incidences of different age categories over China available by the Chinese National Cancer Center^4^,^57^. In order to reduce unstable incidence estimation at the township level (which could be caused by small populations) the 5-year averages of TC and BC incidences were calculated and used to assess the co-existence between the two kinds of cancers (the person-years for each township were estimated by multiplying their 2010 female population by 5).

### The Integrative Disease Predictability (IDP) Method

Modeling the association between TC and BC incidence distributions involves considerable uncertainty due to limited knowledge of the underlying mechanism varying across space-time (epistemic uncertainty) and the complexity of the phenomenon itself in multiple space-time scales (ontic uncertainty). However, if the randomly varying TC and BC incidence distributions are modeled as *spatiotemporal random fields*, S/TRF^58^, it is possible to evaluate the statistical space-time properties of these distributions. Therefore, in this work the TC and BC distributions were mathematically represented as the S/TRF


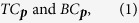


respectively, where ***p*** = (***s**,t*) is a space-time vector denoting spatial location ***s*** and time instant *t*. The space-time domain of the diseases is a continuum, in which space represents the order of co-existence and time represents the order of successive existence of TC and BC. Randomness manifests itself as an ensemble of possible realizations regarding the TC and BC incidence distributions. Methodologically, a disease random field is then a collection of realizations (possibilities) of the cancer distribution, where the probability that each one of these possible realizations occurs is expressed by the corresponding probability law of the disease (see, e.g., *f*(*BC*) in Eqs (7, 8) below). As it turns out, incidence distributions are well represented by theoretical probability laws (Gaussian or non-Gaussian), and this permits us to calculate various space-time properties of these distributions with reasonable accuracy.

In the random field theory context, the IDP approach^52^,^53^,^59^ considers the study of the TC-BC co-existence across space-time as a knowledge synthesis affair that leads to an association model integrating different kinds of cancer data in order to draw sound inferences about the health situation in Hangzhou city. Since the TC-BC association is an uncertain affair, mainstream deterministic logic is of little use. Stochastic inference, on the other hand, by definition has the flexibility for a realistic description of disease associations. The IDP approach postulates that the existence of a TC-BC association should lead to actual BC prediction errors 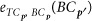 at a set of “control” points ***p**'* ≠ ***p*** when data about both TC and BC are combined that are consistently smaller than the BC prediction errors 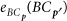 when only BC data are used, i.e.,


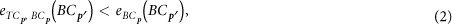


where space-time “control” points are locations-times where BC data are available and can be compared with BC incidence predictions.

The predictability criterion of Eq. (2) may take many forms. A possible form is provided by the *IDP coefficient* defined as the prediction error difference^53^


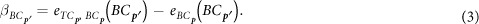


Then, a consistently negative 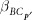 map supports an association between BC-TC incidence distributions. The concept underlying Eq. (3) is straightforward: if BC incidence predictability is consistently improved across the space-time disease domain by using valid information about the TC incidence distribution, this is an indication of a TC-BC association. Given the current state of understanding of fundamental processes (biological, genetic etc.), we may not be able to explain this association in rigorous scientific terms, but the TC-BC association nevertheless exists and cannot be ignored. In the present study, for cross-verification purposes we applied the IDP criterion to assess the predictability of both kinds of cancer, i.e., in addition to 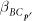, the IDP coefficient


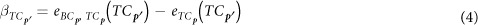


was also calculated, where now 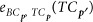 *vs*. 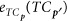 denote, respectively, that information about both TC and BC incidences *vs*. information only about TC incidence was taken into account when TC predictions at the selected control points ***p**'* were generated. Specifically, when one of the two cancer types (TC or BC) was used as hard (i.e., exact) data to calculate the prior incidence probability density functions, the other type (BC or TC) was used as soft (i.e., uncertain) data combined with hard data to predict cancer incidence across space-time. Providing that there are no strong effects due to hidden confounding factors, an improved prediction of the BC (resp. TC) incidence distribution obtained from the combination of TC and BC data as compared to the prediction obtained merely from BC (resp. TC) data supports the existence of an association between the TC and BC incidence distributions.

In practice, the prediction errors at the set of control points ***p**'*, i.e., 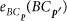, 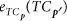, 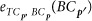, and 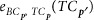, were calculated using scalar and vector BME techniques^60^; i.e.,


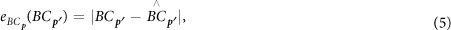


where 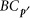 and 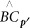 denote, respectively, the actual and scalar BME-predicted BC incidences at the control points ***p**'* using only BC data at points ***p***; and


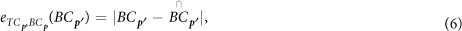


where 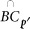 now denote the vector BME-predicted BC incidences at ***p**'* using both BC and TC data at points ***p*** (and, similarly for 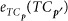 and 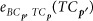).

The basic set of BME equations used in the calculation of the 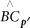 and 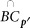 space-time predictions are^51^,^53^,


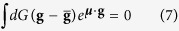



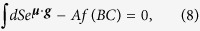


where ***g*** is a vector of functions expressing mathematically the available core knowledge base *G*, including spatiotemporal dependence models, epidemic laws, and scientific theories; 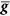 denotes the mean value of ***g***; *S* represents the available site-specific (Hangzhou city) knowledge base about the diseases as described earlier; ***μ*** is a vector of coefficients representing the relative importance of each ***g***-function (***μ***·***g*** denotes the inner product of the vectors ***g*** and ***μ***, which are both functions of space-time); and *A* is a normalization parameter. Eqs (7, 8) can be solved with respect to the BC probability law *f*(*BC*) at all disease mapping points of interest (i.e., space-time points at which BC predictions are sought). Software libraries have been developed dealing with the solution of Eqs (7, 8) in real world conditions, including BMElib, SEKS-GUI, Quantum BME, and StarBME^60^. Eqs (7, 8) are valid in terms of TC and have been used for the calculation of the 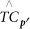 and 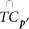 space-time predictions. More technical details concerning the BME equations above can be found in the relevant literature.

BME techniques are successfully used in many scientific disciplines^61^,^62^,^63^,^64^,^65^,^66^,^67^, and among their attractive features are the following^51^,^60^,^68^: they offer a complete disease spread characterization in terms of spatiotemporal random field theory; they account for various sources of knowledge, core and case-specific (scientific models, probabilistic evidence, empirical relationships); they do not require any assumption regarding the shape of the underlying probability law (hence, non-Gaussian laws are automatically incorporated); they lead to nonlinear predictors, in general, and can obtain mainstream predictors (e.g., kriging) as their limiting cases; they can model heterogeneous (spatially non-homogeneous/temporally non-stationary) disease data; and they can perform vector disease mapping (i.e., involving several diseases simultaneously), as is the case with TC and BC incidences in the present study. More details concerning BME theory and techniques can be found in the relevant literature.

**Figure 1:**
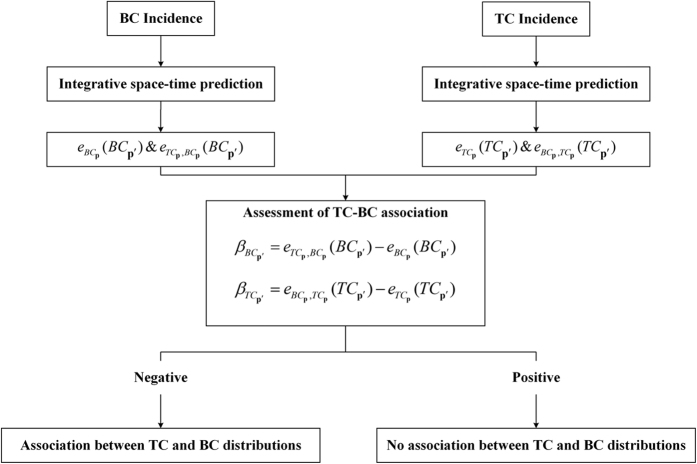
An outline of the IDP approach.

The IDP criterion can provide a useful approach to estimate quantitatively the overall and local strength of TC-BC associations in a region during the time period of interest on the basis of successful space-time cancer incidence predictions (the outline of the IDP approach is given in Fig. 1). In the present Hangzhou study:

- The 200 township level incidence data were considered in a 10-fold validation framework in which the data were divided into 10 distinct subsets (each subset included 1/10th of the total dataset). Then, each one of these (1/10th) subsets was, in turn, selected as the validation set, whereas the remaining subset (consisting of 9/10th of the data) served as the training subset providing the hard data used to estimate incidence rates at the validation points.
- TC (resp. BC) incidence was predicted separately based on its own incidence (hard) data and the TC (resp. BC) prediction error was recorded as 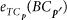 (resp. 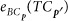).
- Soft incidence data were introduced in the selected validation subset in the form of probability density functions (pdf) constructed by first developing a linear regression model to relate TC and BC incidences (which showed a high linear correlation), and then using the model’s means and variances to construct the TC pdf (resp. BC pdf).
- The TC (resp. BC) incidence was predicted based on its own and the other cancer type data, i.e., when predicting TC (resp. BC) incidence, TC (resp. BC) incidence data were used as hard data (this is the 9/10^th^–data subset above) and BC (resp. TC) data were used as soft data (this is the 1/10^th^ –validation subset) in the form of TC pdf (resp. BC pdf) obtained in (*c*) above; and the prediction error was recorded as 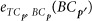 (resp. 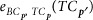).
- Finally the association between TC and BC incidences could be assessed quantitatively on the basis of 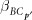 and 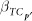. As noted earlier, the TC-BC association at the population level is supported if the 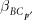 and 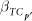 are found to be consistently negative across the space-time study domain.

Computationally, all the data processing and mapping operations were carried out with ArcMap 9.3^69^, whereas BME space-time TC and BC incidence predictions were generated using the SEKS-GUI v1. 0.8 software^60^.

## Results

**Figure 2:**
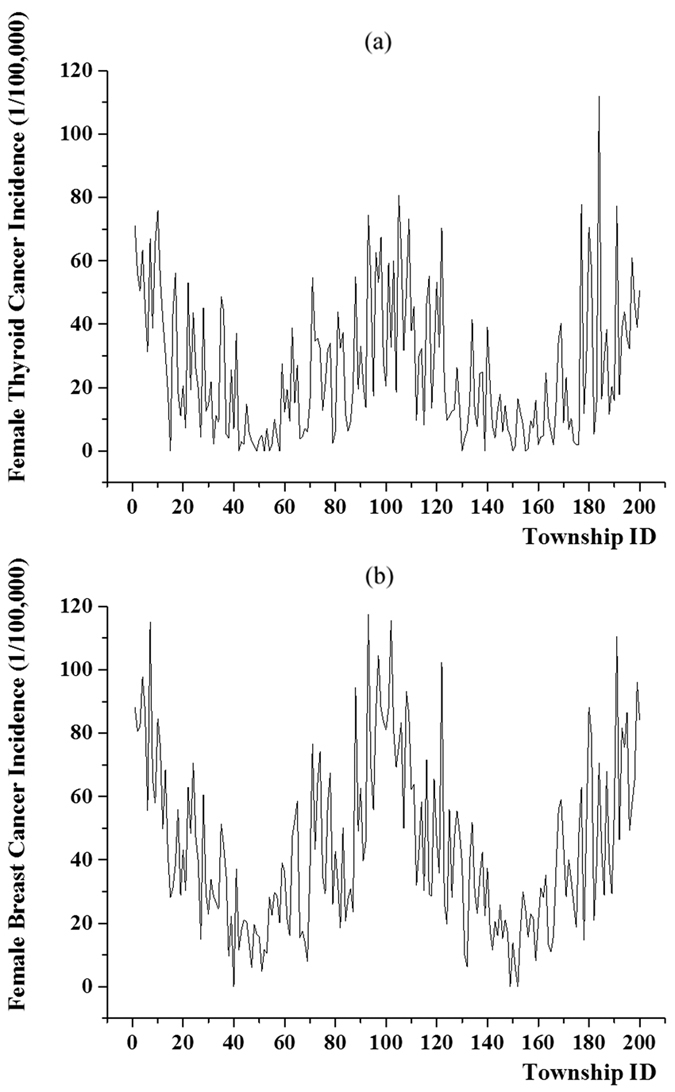
5-year average female thyroid cancer (**a**) and breast cancer (**b**) incidence in each township.

The present work is, in a sense, an ecological study conducted using data at the township level. Naturally, two limited cases were noticed in townships with small populations: the TC and BC incidences would be zero if no cancer patient was recorded during a specific year, and extremely high when for a small population base even a small number of patients would lead to high incidence. As noted earlier, to overcome these computational issues the 5-year averaged TC and BC incidences were calculated to produce more stable incidence estimates. Accordingly, Fig. 2a displays the 5-year averaged profile of TC incidence for the 200 Hangzhou townships considered, and Fig. 2b that of BC incidence. Visual comparison of Fig. 2a,b clearly suggested the existence of a strong correlation between TC and BC incidences. Also, the Pearson and Spearman rank correlation coefficients of the 5-year averaged TC and BC incidences were found to be 0.797 (*p* < 0.01) and 0.808 (*p* < 0.01), respectively, at the township level.

**Figure 3:**
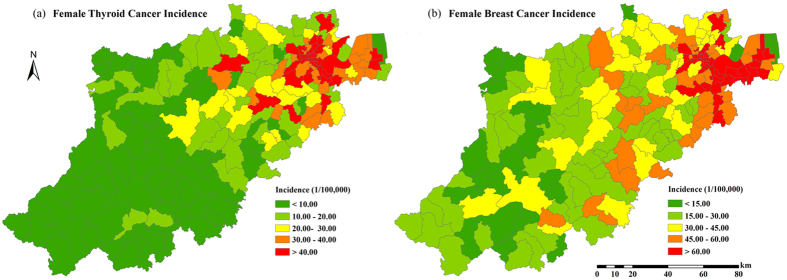
The distribution of 5-year average female thyroid/breast cancer incidence in Hangzhou. (2008–2012). Created by ArcMap 9.3.1 http://www.esri.com/software/arcgis/eval-help/arcgis-931.

In Fig. 3a the geographical TC incidence distribution averaged during the 5-years (2008–2012) exhibited considerable spatial variability across the Hangzhou area: townships located in the southwestern parts of the region revealed the lowest TC incidence (less than 10 per 100,000 people), whereas townships located in the northeastern parts experienced the highest incidence (more than 40 patients per 100,000 people). The pattern of geographical BC distribution averaged during the 5-years (Fig. 3b) was similar to that of TC: the southwest areas showed the lowest incidence (less than 15 patients per 100,000 people), whereas the northeast areas showed the highest incidence (more than 60 patients per 100,000 people).

**Figure 4:**
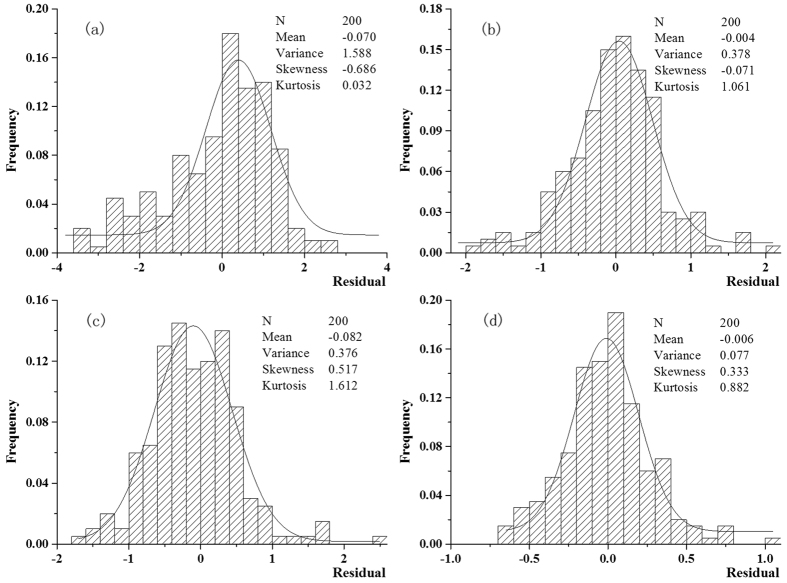
The distribution of prediction errors through Bayesian maximum entropy techniques. (***a***) only TC incidence were used as hard data to predict TC incidence, (***b***) TC incidence were used as hard data and BC incidence were used as soft data to predict TC incidence, (***c***) only BC incidence were used as hard data to predict BC incidence and (***d***) BC incidence were used as hard data and TC incidence were used as soft data to predict BC incidence.

Figure 4 displays the frequency graphs of incidence prediction errors derived by the BME technique in the following cases: (*a*) only TC incidence values were used as hard data to predict TC incidence; (*b*) TC incidence were used as hard data and BC incidence information was used as soft data to predict TC incidence; (*c*) only BC incidence values were used as hard data to predict BC incidence; and (*d*) BC incidence values were used as hard data and TC incidence information was used as soft data to predict BC incidence.

In particular, the predictive TC incidence map corresponding to the prediction error frequency graph of Fig. 4b had a smaller root mean square error, (RMSE = 5.21) than that corresponding to Fig. 4a (RMSE = 16.27). And, the TC prediction error graph of case *b* was more Gaussian-like than that of case *a*. The comparison of cases *a vs. b* clearly shows that the incorporation of soft BC incidence data provided an improved TC incidence prediction compared to that using hard TC incidence data only. Similarly, the frequency graph of Fig. 4d displayed a smaller RMSE (0.74) than that of Fig. 4c (RMSE = 5.78). Also, the TC prediction error graph of case *d* was more Gaussian-like than that of case *c*. Again, the comparison of cases *c vs. d* demonstrated that the incorporation of soft TC incidence data provided better BC incidence predictions compared to that using only hard BC incidence data.

**Figure 5:**
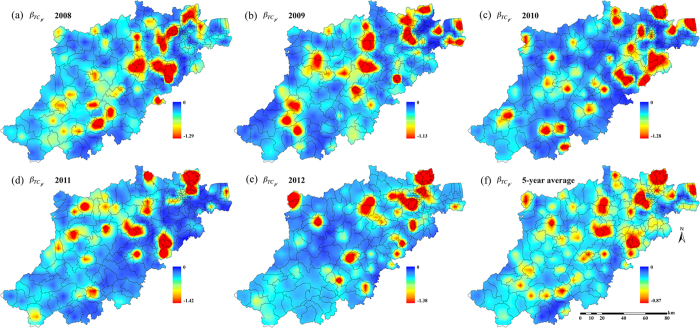
Maps of 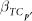. The 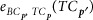 denotes TC incidence prediction errors obtained using TC incidence as hard data and BC incidence as soft data; and the 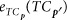 denotes TC incidence prediction errors obtained using TC incidence as hard data only. Created by ArcMap 9.3.1 http://www.esri.com/software/arcgis/eval-help/arcgis-931.

**Figure 6:**
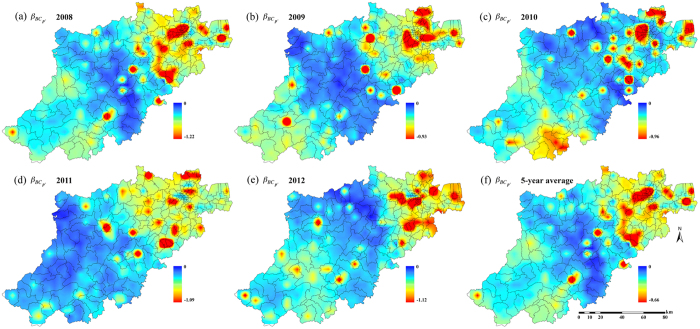
Maps of 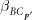. The 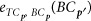 denotes BC incidence prediction errors obtained using BC incidence as hard data and TC incidence as soft data; and the 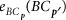 denotes BC incidence prediction errors obtained using BC incidence as hard data only. Created by ArcMap 9.3.1 http://www.esri.com/software/arcgis/eval-help/arcgis-931.

Figures 5 and 6 show the distribution of 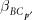 and 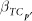 calculated by Eqs (3) and (4). Specifically, in Fig. 5a–e we see the maps of the coefficient 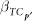 in Hangzhou during the years 2008–2012, whereas Fig 6a–e show the maps of the coefficient 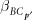 in Hangzhou for the same time period. Note that the values of 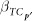 and 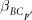 were consistently negative across the Hangzhou area during the 5-year period, which, according to the IDP criterion, implies the presence of a “TC incidence-BC incidence” association at the local township population level during the 5-year period. Since the IDP coefficients provide a measure of the geographical variation of the contribution of the TC-BC incidence association to incidence prediction, the higher (in absolute value) the coefficient 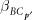 (resp. 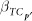) in an area, the higher the contribution of the TC-BC association in BC (resp. TC) incidence prediction in this area. This contribution varies across space and time, and it can be different for the two cancer types. For example, Fig. 5a reveals that the contribution of the TC-BC association in TC incidence prediction is higher in parts of southwestern Hangzhou, whereas in Fig. 6a the association’s contribution in BC incidence prediction is higher in parts of northeastern Hangzhou. Figures 5f and 6f show the 5-years averaged maps of 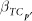 and 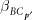, respectively.

**Figure 7:**
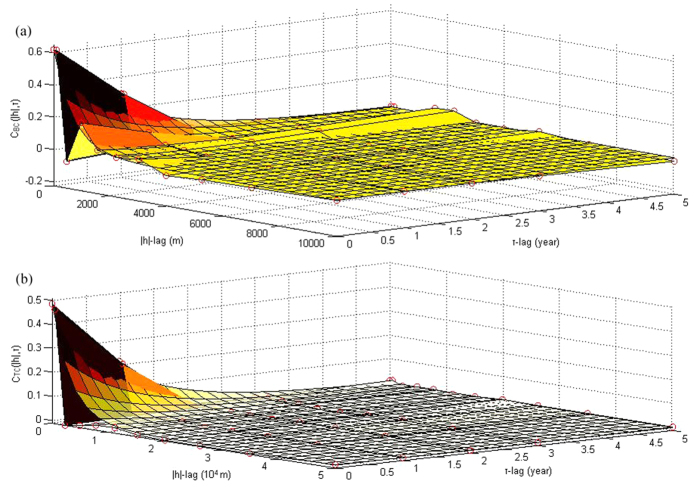
Plots of the space-time covariances of the TC incidence and BC incidence distributions, (***a***) 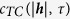, and (***b***) 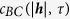, respectively. Circles denote experimental (sample) covariance values, whereas continuous (color) surfaces and continuous lines denote the fitted theoretical models.

Interestingly, the geographical variations of the 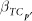 and 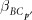 distributions of each one of the five years considered (Figs 5a–e and 6a–e) showed some small differences, which were due to corresponding differences in the disease prediction error maps (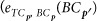 *vs*. 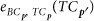), which, in turn, were due to the different TC and BC datasets as well as to the different space-time covariances, 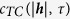 and 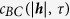, used in TC and BC incidence prediction. Indeed, the different shapes of the two space-time incidence covariances can be clearly seen in Fig. 7a,b (the spatial TC correlation range is much longer than the BC correlation range, implying stronger geographical dependence between TC incidences than between BC incidences, the TC incidence variance is smaller than that of BC incidence, implying less local TC variation than BC variation, etc.). These differences between the incidence covariances can affect the results of BME prediction of the TC and BC incidences across space-time.

Moreover, the global numerical values of the IDP coefficients, i.e. the averages of the 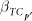 and 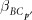 coefficients for the five maps of Figs 5a–e and 6a–e, can be calculated by, respectively,


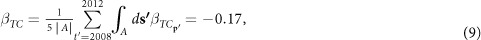



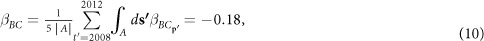


where 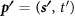, *A* denotes the Hangzhou area, and |*A*| is the magnitude of *A* (a total of 1000 space-time points were used in the calculation of the averages in Eqs (9, 10). The *β*_*TC*_ and *β*_*BC*_ values, which provide a quantitative assessment of the magnitude of the TC-BC association, were found to be practically identical, as should be expected, since both coefficients refer to the same TC-BC association. The result of Eqs (9, 10) further support the spatiotemporal co-existence of TC and BC incidence distributions at the population level, according to the IDP criterion, and are in agreement with some earlier findings suggesting the co-existence of TC and BC diseases^17^,^39^,^40^,^41^.

## Conclusions-Discussion

Since BC and TC are two of the most common cancers in the female populations, it is important to study whether the two cancers co-exist at the administrative level. The quantitative assessment of this association over a geographical region and during a specified time period is an important but difficult endeavor. Indeed, epidemiologic research concerning the spatiotemporal co-existence of diseases involves a number of traditional methods (visual comparisons of disease patterns, statistical analysis of disease occurrences across a geographical region, multi-perspective analysis, and geographical information systems techniques) that have been found to have certain drawbacks when seeking to identify disease interactions and causation. For example, previous studies have used correlation analysis to assess any similarities and differences between BC and prostate cancer (PC) mortalities in Spain, but they lacked the ability to assess BC-PC associations at specific locations and time periods^7^. Analogical, cluster maps and correlation analysis were also used to compare the spatial trends of BC and PC incidences in the United States^55^. Although the results could determine whether or not the two cancers are “high-high” or “low-low” correlated in specific geographic areas, they still lacked the ability to assess local correlations quantitatively.

In the present work, the implementation of the IDP criterion to evaluate the spatiotemporal co-existence of BC and TC incidence distributions offered new insight into disease associations in Hangzhou city during a 5-year time period. The theoretical support of the IDP criterion is twofold: mathematically, the combined structural and uncertain components of TC and BC incidence distributions are represented by the spatiotemporal random field theory; and, logically, the potential TC-BC associations are assessed in terms of consistent improvements in the incidence predictability of one disease resulting by the synthesis of information sources about both diseases. It should be noticed that the IDP approach can gain additional support from the fact that in health sciences one finds an interesting connection between prediction and causation: occasionally, the same epistemic and empirical conditions that generate sound predictions of a disease are used to obtain meaningful causal explanations of it, as well^43^.

A significant positive correlation was observed between the TC and BC incidence distributions in Hangzhou city during 2008–2012. Subsequently, the magnitude of the association between space-time TC and BC distributions, at the global and local scales, was quantitatively assessed in terms of the IDP coefficients. The results supported the spatiotemporal co-existence of TC and BC incidence distributions at the population level. They also showed that the Hangzhou population had a significantly higher risk to develop TC when an increased BC incidence had been observed in the area during the 5-year time period, and vice versa.

The spatiotemporal co-existence of TC and BC could be partially attributed to common risk factors. First, as is well-known, hormonal, menstrual and reproductive factors (early age at menarche, later age pregnancy and menopause, increased exogenous hormone use and hormone replacement therapy etc.) have a strong effect on BC risk, and certain studies have hypothesized that the same factors may play a role in TC etiology, as well. These studies, however, are not consistent: for example, a case-control study in the Middle East has suggested that reproductive factors may influence or contribute to female TC risk (although hormone use was not associated with female TC risk)^70^, whereas a prospective study of Norway women during 1961–1989 suggested that there was no strong association between reproductive factors and TC risk^56^. In recent years, a population-base, case-control TC study among young women in France pointed out that reproductive and hormonal factors leading to estrogen exposure can increase TC risk^39^, but the European Prospective Investigation into cancer and Nutrition (EPIC) cohort study suggested that hormonal use, reproductive and menstrual factors have no strong associations with TC etiology^18^. In the present study, female BC incidence showed a similar geographical pattern with female TC incidence, e.g., the incidences of both diseases gradually increased from the southwestern to the northeastern part of Hangzhou. It was also established that higher (lower) BC incidence was co-existence with higher (lower) TC incidence, and the TC-BC incidence associations were consistent across the study area. These findings supported the hypothesis that hormonal, menstrual and reproductive factors are associated with TC risk.

Second, TC incidence revealed a sharp increase in recent decades, however this increase has been detected mainly in small (<2 cm), well-differentiated papillary cancers, whereas TC mortality did not show a significantly increasing trend. Therefore, improved detection of subclinical diseases that are indolent and would never cause symptoms (overdiagnosis) due to the increasing use of more sensitive diagnostic methods could, at least partially, explain the increasing TC incidence^34^,^71^. In previous ecological studies, the socioeconomic status was used as a surrogate of access to health care (e.g., health insurance coverage) and to diagnostic technologies (e.g., imaging and fine-needle aspiration)^72^,^73^. It has been found that better socioeconomic status (representing better health care and diagnostic technology accessibility) was positively correlated with TC risk, and this finding is also valid in the BC case. In fact, many studies have discussed the association between socioeconomic status and BC risk^2^,^11^,^54^,^74^. Higher socioeconomic status (assessed in terms of higher income, education level and less unemployment) represents better health awareness and health care accessibility that could lead to BC be diagnosed at an early stage, thus leading to positive correlations between BC and socioeconomic status. The co-existing TC and BC cancer types were characterized by 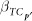 and 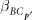 distributions that were both spatially heterogeneous at the township level (Figs 5 and 6). Compared to previous Hangzhou studies^54^, the stronger TC-BC co-existence was observed mainly in subdistrict areas with the highest socioeconomic status. Better health care, diagnostic technology accessibility and over-diagnosis assessment might strengthen the co-existence of TC and BC, which could be also the focus of future works.

Third, there are some other common TC and BC risk factors that may be responsible for the spatial coexistence of the two cancers, such as urban-rural disparity, higher BMI, smoking and alcohol consumption, exogenous estrogen exposure (PCBs, PBDEs, estradiol, oestrone). The TC and BC pathogeneses are very complex. Although BME and IDP could control for confounding factors, due to the ecological design of the present study and data limitations we did not control for these factors. Other study limitations included the lack of specific information about TC sub-types (papillary thyroid carcinoma, follicular thyroid carcinoma etc.) and BC sub-types (estrogen receptor positive −ER+, estrogen receptor negative −ER− etc.). As there are different etiologies for each cancer sub-type, the lack of sub-type information may create some problems in disease etiology. Although townships were sufficiently populated for the present study purposes (so that, e.g., hard incidence data can be considered in the Hangzhou study with high confidence), some techniques (including BME^64^ and Poisson kriging^75^) can model uncertain incidence information, thus improving incidence estimation accuracy. This is a possibility to be explored in a future work.

## Additional Information

**How to cite this article**: Fei, X. *et al*. Spatiotemporal Co-existence of Female Thyroid and Breast Cancers in Hangzhou, China. *Sci. Rep.* **6**, 28524; doi: 10.1038/srep28524 (2016).
